# Expert-Led Module Improves Non-STEM Undergraduate Perception of and Willingness to Receive COVID-19 Vaccines

**DOI:** 10.3389/fpubh.2022.816692

**Published:** 2022-05-18

**Authors:** Christina N. Morra, Sarah J. Adkins-Jablonsky, M. Elizabeth Barnes, Obadiah J. Pirlo, Sloan E. Almehmi, Bianca J. Convers, Derek L. Dang, Michael L. Howell, Ryleigh Fleming, Samiksha A. Raut

**Affiliations:** ^1^Department of Biology, University of Alabama at Birmingham, Birmingham, AL, United States; ^2^Alabama College of Osteopathic Medicine, Dothan, AL, United States; ^3^Department of Biology, Middle Tennessee State University, Murfreesboro, TN, United States

**Keywords:** COVID-19, COVID-19 vaccine, guest lectures, vaccine awareness, non-STEM majors, non-majors, biology

## Abstract

As evidence mounted that existing prevention methods would be insufficient to end the COVID-19 pandemic, it became clear that vaccines would be critical to achieve and maintain reduced rates of infection. However, vaccine-hesitant sentiments have become widespread, particularly in populations with lower scientific literacy. The non-STEM major (called non-major) college students represent one such population who rely on one or more science classes to develop their scientific literacy and thus, become candidates of interest for the success of the COVID-19 vaccine campaign. As these students have fewer opportunities to learn how to identify reputable scientific sources or judge the validity of novel scientific findings, it is particularly important that these skills are included in the science courses offered to non-majors. Two concurrent non-major biology courses (*N* = 98) at the University of Alabama at Birmingham in Spring 2021 completed Likert questionnaires with open-ended questions prior to and after an expert-led Vaccine Awareness educational intervention addressing vaccine-related concerns. In the module, experts gave presentations about COVID-19 related to microbiology, epidemiological factors, and professional experiences relating to COVID-19. Ten students agreed to participate in post-semester one-on-one interviews. Student interviews revealed that students perceived guest lecturers as providing more information and assurance. Questionnaire data showed an increase in student willingness to accept a COVID-19 vaccine as well as increased student perception of the COVID-19 vaccines as both safe and effective (Wilcoxon Rank Sum Test, *p* < 0.05). However, the questionnaire data revealed 10 of 98 students remained vaccine-resistant, and these students expressed insufficient research and side effects as leading vaccination concerns. Overall, we show expert-led modules can be effective in increasing non-majors willingness to accept COVID-19 vaccines. Future research should explore the experiences of non-majors and guest lectures, particularly as they relate to vaccination and vaccine concerns.

## Introduction

The higher education shutdown in Spring of 2020 due to COVID-19 permanently changed the landscape of education (1), placing a considerable burden on nearly all aspects of educational routines (2). According to UNESCO, as of 1st April 2020, schools and higher education institutions were closed in 185 countries, affecting nearly 90% of total enrolled learners (3). Many college students across the world had to adapt to a remote learning mode due to the COVID-19 pandemic (4, 5). While the majority of academic institutions shifted to distance learning, colleges and universities that continued in-person instruction were found to be associated with increased incidence of COVID-19 cases (6). In order to reduce the transmissibility of this virus, most university campus administrations instituted preventive measures such as screening for COVID-19 prior to starting the semester (7) and mandating facial masking (8). While these measures were evidence-based, vaccination became recognized by Spring 2021 as the most effective preventative measure in reducing risk of COVID-19 infection and death (9).

Several COVID-19 vaccinations have been approved by the Food and Drug Administration since the eruption of the pandemic including those produced by Pfizer-BioNTech (10, 11), Moderna (12) and Johnson & Johnson (13). Despite the wide availability of these vaccines to the public, the vaccination rates remain low and have not yet met the herd immunity threshold (14, 15). The second wave of COVID-19 that emerged during 2021 led to a major shift in the public perception about vaccination (16). In fact, it is becoming increasingly accepted among the public that widespread vaccination is the most effective method to control the COVID-19 pandemic (17). However, despite the publicly available vaccines and the shift in the public perception, a large percentage of eligible people remain unvaccinated, with more than 45% of US adults not fully vaccinated (indicating two doses of a 2 sequence vaccine or one dose of a 1 sequence vaccine) as of March 2022 (9). We note that ongoing research shows that natural immunity provides defense for several months amongst people who have previously been infected with COVID-19, however natural immunity from infection as a sequela does not prevent an initial infection (18).

Even still, several factors have been proposed to explain the lower-than-expected vaccination rate.

These factors are related in part to (a) perceived issues of vaccine efficacy and its safety profile and (b) lack of trust in both the scientific community and the location of vaccine development (15, 19, 20). Of particular importance is the rampant vaccine mistrust to be higher among non-white and non-male demographic groups (20–23). Furthermore, both lower education and information levels were found to be correlated with lower willingness to get vaccinated and to abide by preventive measures (24, 25). Such diverse population of individuals include non-STEM majors (henceforth called “non-majors”) college students who may depend on a college science curriculum to enhance their scientific literacy (26). Non-majors differ from STEM majors and these differences make further investigation well-warranted. First, due to their limited exposure to science in general, or to the rigor of scientific methodology, non-major students tend to have a lower trust and confidence in the scientific community and their recommendations (27) and thus may decide to seek information related to vaccines and other topics of interest from social media (28, 29). This in turn can lead non-majors to be further prone to conspiracy beliefs related to topics like vaccination (30). Second, this group tends to be more diverse demographically and prefers more personailzed pedagogies (31, 32). Third, there are up to three times as many articles (accessible via Google Scholar) about STEM majors than non-majors (data not shown), a disparity which highlights the lack of research related to non-majors' biology curriculum. These characteristics necessitate a better understanding of the internal dynamics of the non-major college students and a targeted approach to overcome the barriers and misconceptions regarding COVID-19 vaccination. There is a clear need to increase evidence-based pedagogy for non-major classrooms, particularly as it relates to decreasing vaccine hesitancy. Based upon this rationale, we invited expert guest lecturers from an array of fields who could speak about COVID-19 vaccines to our non-majors biology students.

A targeted approach should include the dissemination of trusted information of the vaccine development, its side effects, profile, and the rate of potential protection via immunity (19, 20, 33, 34). While the practice of inviting guest lecturers seems fairly ubiquitous in higher education, the recommendation to invite guest lectures is primarily anecdotal (35). The limited research that exists in the use of expert guest lectures to impart this type of key information suggests that the perceived relevant experience of expert guest lecturers increases the impact of the lecture (35–39). We have previously shown in an experimental study that non-major students are receptive to virtual expert-led guest lectures about COVID-19 (40), but have yet to explore how views can change related to the COVID-19 vaccines especially through a pre-post design. Here, we investigated the degree to which non-majors' attitudes shift after our intervention with attention to the following questions:

1. How did non-major perceptions of the COVID-19 vaccine change pre to post intervention?
2. How did non-major students' intention to get a COVID-19 vaccine change pre to post intervention?
3. What were non-major students' perceptions of the COVID-19 guest lectures?
4. What are remaining reasons vaccine-resistant students reject COVID-19 vaccines?

## Methods

### Study Population

Participants in this population were all students at The University of Alabama at Birmingham (UAB), which serves as the largest research-intensive university in Alabama, a state in the southeastern United States. Participants were non-biology major students taking one of two biology courses: Topics in Contemporary Biology (BY101) or Introduction to Microbiology (BY 261). Courses are 3 credit-hour non-major courses remotely taught by author S.R. during the Spring 2021 semester. Topics in Contemporary Biology is a generic education requirement for science enrolling 97 non-major students in Spring 2021 with no prerequisite. Grading for the lecture course (300 total points) was as follows: two exams (100 points each), pre-COVID-19 Vaccine Awareness questionnaire (10 points), post-COVID-19 Vaccine Awareness questionnaire (10 points), Service-Learning project (60 points), pre-Service-Learning reflection (10 points), and post-Service-Learning reflection (10 points). Service-Learning was assigned following this module and post-questionnaire and therefore was not expected to influence student responses herein. Forty three students in this course consented to take part in this research (44% participation rate). Introduction to Microbiology is a required prerequisite for nursing majors and enrolled 216 students in Spring 2021 with one required prerequisite, which could include BY101. The course required enrollment in a lab course. Grading for the lecture course (220 total points) was as follows: two exams (100 points each), pre COVID-19 vaccine questionnaire (10 points), and post COVID-19 vaccine questionnaire (10 points). Students were able to increase their grade by an additional 10 points through bonus quizzes. One hundred and twelve students in this course consented to take part in this research (52% participation rate) for a total of 155 UAB students consenting to take part in this research. Demographic information and response rate is available in Supplementary Table 1. This research study was approved by the University of Alabama at Birmingham IRB, Protocol number: IRB-300006871.

### Questionnaire

Participants in both courses completed the same pre and post questionnaires, completed as a quiz through the course's online learning management system. The pre and post questionnaires were identical (see Box 1). Questions were edited from questions included in the Pew Research Center's 2020 American Trends Panel, Wave 79 survey conducted November 18-29, 2020, which polled 12,648 individuals in the United States of America (41) and/or were developed by C.M., S.A-J. and S.M. for the purposes of this study. The wording of the questions used in this study were determined as readable and clear by a focus group of 5 UAB students before they were distributed for this study. “Note response choice “NA” was an artifact recommended by the focus group to account for participants who may have been vaccinated. While most participants did not choose “NA”, “NA” was taken into account during the analysis." All students enrolled in the courses were instructed to complete the questionnaire as part of the graded assignments for their course grade. Only the responses of students who confirmed they watched the intervention module and consented to be included were analyzed.

Box 1 **COVID-19 Vaccine Awareness questionnaire.**

Introduction: This assignment aims to address COVID-19 concerns you may have to improve your classroom experience for a targeted intervention on the safety and effectiveness of the COVID-19 vaccines. Before 1/21/21 complete this assignment.

1. Have you received a COVID-19 vaccine, or do you intend to get a COVID-19 vaccine?
  Yes, I have received two doses of a COVID-19 vaccine
  Yes, I have received the first dose of a COVID-19 vaccine
  No, but I will—I DO intend to get the vaccine
  No, but I will not—I DO NOT intend to get the vaccine
Prefer not to answer

2. I have enough information to make a decision regarding accepting the COVID-19 vaccine.
  5-point Likert with additional “NA—I have already been vaccinated by one or two doses”
3. I will accept the COVID-19 vaccine if my school or employer mandated it.
  5-point Likert with additional “NA—I have already been vaccinated by one or two doses”
4. The coronavirus outbreak is/has been a risk to my personal health.
  5-point Likert with additional “NA—I have already been vaccinated by one or two doses”
5. If you or someone you know has been affected by COVID-19, how has that impacted your willingness to get a COVID-19 vaccine?
  Open ended
6. The approved COVID-19 vaccines are safe.
  5-point Likert with additional “NA—I have already been vaccinated by one or two doses”
7. The approved COVID-19 vaccines are effective.
  5-point Likert with additional “NA—I have already been vaccinated by one or two doses”
8. What are the 3 major reasons why you will or will not accept a COVID-19 vaccine? Please list them below in the order of priority.
  Open ended

Students had the first week of class to complete the pre-questionnaire. One hundred and fifty five consented students completed the pre-questionnaire. After students developed expertise during the 3-week module (described below), students had 1 week to complete the post questionnaire. One hundred and fifty four consented students completed the post questionnaire.

### Development of Expertise—Vaccine Awareness Module

These modules, like previous modules implemented at UAB (40, 42) used the framework of backwards design (43). The course learning objectives relevant to this module were: “Understand the basic process of science and identify the valid sources of scientific literature” and “Analyze and apply scientific information to make everyday decisions.” From these objectives, a Service-Learning assessment was created (this assignment was completed after this module and is beyond the focus of this manuscript), and based on the objectives and assessment, the expert lecturers and Q&A were chosen as the most suitable learning activity. The expert Vaccine Awareness Module included two pre-recorded sessions (which were both required to be watched) and two live sessions (which were required to be watched live or via recording). Additionally, there were two pre-recorded lectures uploaded to the course learning management system related to the basic microbiology of viruses. This module was completed over 3 weeks between the end of January and early February of 2021. The module included:

1. Pre-recorded session: “Basics of Vaccines” (S.R., PhD in Biology, instructor of record)
2. In this lecture there was a discussion about microbiology of viruses, disease transmission, epidemiology, and general immunological principles.
3. Pre Recorded session: “COVID-19: The Virus, the Disease and the Vaccines” (C.M., PhD in Microbiology)
4. In this lecture, SARS-CoV-2 virology, COVID-19 symptoms, infection rates and mutants were covered. The different COVID-19 vaccines being studied were compared for what they contain, how they work, vaccine dosage and schedule, and, when the data was available, what their efficacy is as well as: how and why masks work, history of medical inequities, and specific COVID-19 vaccine misinformation.
5. Live session: “Demystifying Vaccine Hesitancy” (Bertha Hidalgo, PhD in Public Health, MPH)
6. In this live session, basic epidemiology, and effective vs. ineffective pandemic cessation strategies were presented. Student questions were also answered.
7. Live session: “Demystifying Vaccine Hesitancy Q&A” (Ellen Eaton, MD clinician in infectious disease)
8. In this session, after introducing her experience as a physician treating COVID-19 patients in the hospital, E.E. answered student questions about the clinical manifestations of COVID-19.

PowerPoint presentations are available upon request.

### One-on-One Interviews

To supplement the information collected in the COVID-19 questionnaires, consented students were invited to participate in one-on-one student interviews via UAB-hosted Zoom meetings. Ten students chose to participate in these interviews: seven from Topics in Contemporary Biology (BY101-2C) and three from Introduction to Microbiology (BY261-1E). The interviews followed a semi-structured format guided by the questionnaire and took roughly 20 minutes per student. The full question script can be found in Supplementary Box 1. Interview transcripts were checked for accuracy by OJP before the transcripts were coded.

### Coding of Interview Responses

The coding approach used a combination of deductive and inductive analyses. This approach was selected as the deductive element allows the research to be guided by the research questions while the inductive element allows for the exploration of the data within the confines of the research questions (42, 44). Three individual coders, S.E.A, V.C., and S.M. separately read the transcript of the 10 interviews. The coders separately devised open coding subthemes (inductive) that broadly applied to the theme of “Guest Lecturers” (deductive). Coders next discussed their respective themes and came to a consensus regarding refined sub themes that were mutually agreed upon by all three coders.

### Coding of Open-Ended Questionnaire Response

The post questionnaire included the open-ended question “What are the three major reasons why you will or will not accept a COVID-19 vaccine? Please list them below in the order of priority.” This question was coded inductively by C.M. and S.A-J.

### Quantitative Analysis

Students were asked prior to their post-Vaccine Awareness Module questionnaire if they had completed the module. Students who did not respond to this question or who responded “no” were not included in this analysis, which in the end included 30 students from BY101 and 68 students from BY261 (*N* = 98). Differences in changes in perceptions of COVID-19 vaccine perceptions from the questionnaire responses were analyzed using R (45). First, a non-parametric Wilcoxon Rank Sum Test was used to compared pre and post scores for Questions 2 (having enough information), 3 (would take vaccine if employer mandated), 4 (virus is a risk to personal health), 6 (vaccine is safe), and 7 (vaccine is effective). To assess the effect of class, we used linear models with “Class” (BY101 or BY261) as a predictor variable for each of the questions (e.g., “post-score~pre-score + class”). Ordinal regression, often used with ordinal numeric data, was used to confirm the degree which “Class” influenced any of the outcomes (e.g., ”post-score~pre-score + class”). The statistical hypotheses were that pre and post values were not equal. Complete RStudio code is available in Supplementary Files Complete R Code.

## Results

**Figure 1:**
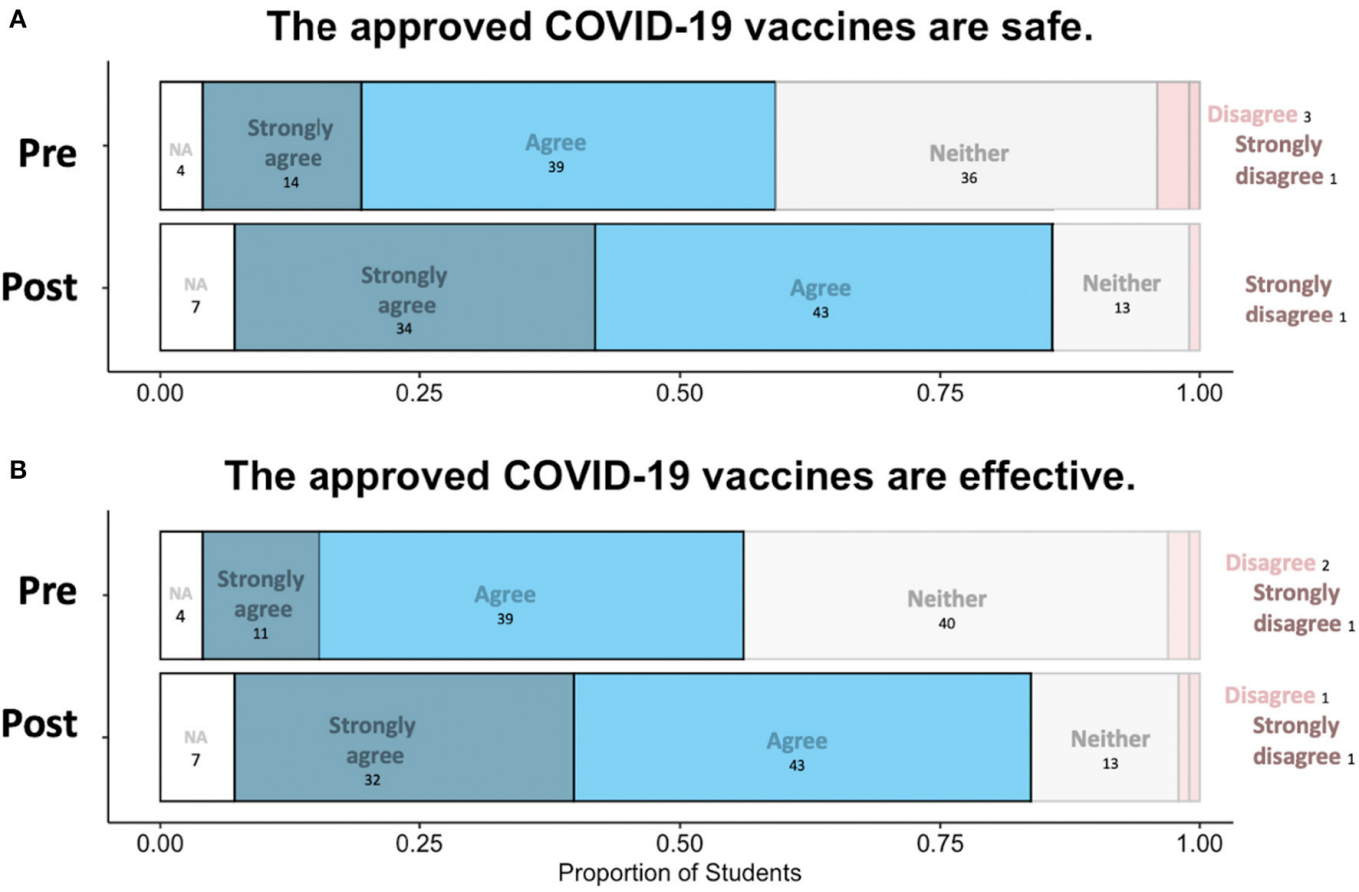
Pre and post agreement to COVID-19 vaccine safety **(A)** and efficacy **(B)**. Pre indicates prior to the module and post indicates perception following module completion. Student agreement ranges from strongly agree (left most and dark blue) to strongly disagree (right most and darker pink). Students responded “NA” if they did not want to answer based on having already been vaccinated. The proportion of total students is on the X axis, where *N* = 98.

**Figure 2:**
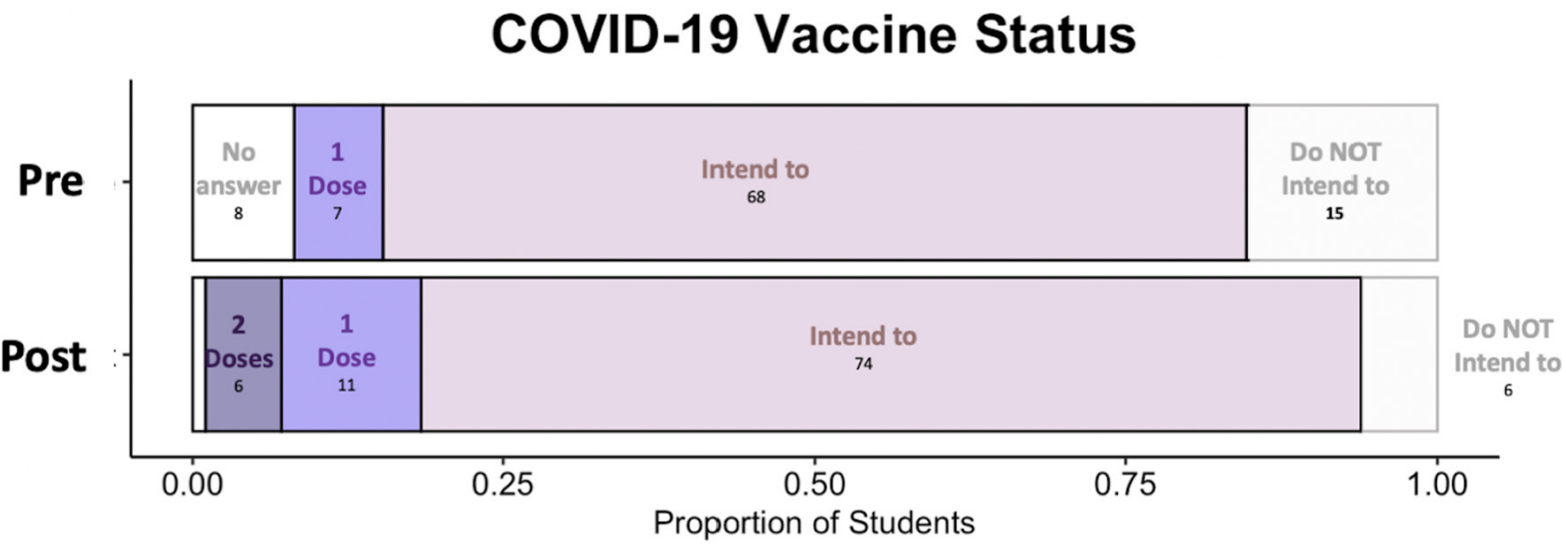
Pre and post COVID-19 vaccination status. Pre indicates prior to the module and post indicates perception following module completion. Student agreement ranges from having two doses of the vaccine (left most and darker purple) to not having any intention of vaccination (right most and lighter). Some students opted to report “Prefer not to answer.” The proportion of total students is on the X axis, where *N* = 98.

Analyses indicated an increase in the number of surveyed students who perceived the approved COVID-19 vaccines as both safe (Figure 1A) (Wilcoxon Rank Sum Test, *W* = 7435.5, *p* < 0.05 and LME, *p* < 0.05) and effective (Figure 1B) (Wilcoxon Rank Sum Test, *W* = 6998, *p* < 0.05 and LME, *p* < 0.05) pre to post module. Additionally, surveyed students were more likely to state that they could make an informed decision regarding vaccination after the completion of the module (Wilcoxon Rank Sum Test *W* = 2529.5, *p* < 0.05, LME, *p* < 0.05). There was no effect of class enrollment on any Likert question outcome (LME, Ordinal Regression). With regards to the question “Have you received a COVID-19 vaccine, or do you intend to get a COVID-19 vaccine?” on the questionnaire, 15 students did not intend to prior to the module and 6 did not intend after the end of the module (Figure 2). In other words, 9 surveyed students changed their mind about receiving the COVID-19 vaccine after completing the Vaccine Awareness Module.

All the 10 interviewed students explicitly mentioned they felt either more informed (*n* = 9) or more assured (*n* = 5) about getting the COVID-19 vaccines because of the guest lectures. Four interviewed students explicitly mentioned the guest lectures had a role in their decision to get vaccinated including indications the expert's provided information as it was the expert's job to understand things about COVID-19 vaccine science (Table 1). Of the interviewed students who directly compared the guest lectures to being taught exclusively by their professor, more expressed a preference for having guest lectures.

**Table 1:** Interview codes related to guest lectures.

**Subtheme**	**Representative quote**	**# of interviews**
**Students felt more well-informed regarding the vaccines after the lectures than before the lectures**	“*It definitely gave you a more a better understanding of like what the vaccine does and like how it works.”*	**9**
**The guest lectures increased their assurance of the vaccine**	“*Saying if you were in a room full of COVID patients that were like positive, that don't mean you can't get COVID, but it just means that you can fight off the symptoms way faster. So that made me feel better, it made me just feel better because like I said like I've seen it all how people were affected by it. So, it's like it made me feel more safer, by them explaining to me that it would just fight off the symptoms more”*	**5**
Guest lectures gave students factual evidence to support their position on COVID-19 vaccination	*: “… you could say that kind of affected my opinion because I was able to kind of back up my opinion with like some facts about you know why I thought it was this way and—Why—it kind of just—got a better glance on my opinions.”*	4
Students were influenced to receive the vaccine after listening to the lecturers	“*I was still kind of a little hesitant, but kind of listening to them talking about it and like all what happens with it, it kind of shifts my thinking like okay, you know, this is something that is a necessary thing to get back to normal…”*	4
Guest lectures eased their concerns regarding the vaccine	“*… they made it kind of less scary, especially since people are always like oh my God this one person got the shot and then they had an allergic reaction, it was the worst thing ever, you should never get it. And then they were just like yeah well that happens with a lot of shots because people are allergic to stuff so definitely help like destigmatize it, I think.”*	4
Students felt it would not have been any different hearing it from their professor, but they were impacted by the guest lectures and/or thought it was nice to hear from more than one person	“*…they impacted my perspective, because they are, I guess, esteemed individuals in our field, and they really understand the topics that they're presenting, and they have studied the topics they were presenting … I do not at all think it would be any different… She taught very well, and I trusted her judgment a lot.”*	4
Students preferred and/or were more interested to hear from an expert guest lecturer rather than the professor	“*Definitely because they're more specialized in their field, I feel like people will obviously you know, listen to the professor, but when it's coming from you know somebody that it's their job to know about this stuff—they're able to not really trust the information more but definitely pay attention to it and really listen to what they're saying.”*	3
Students felt indifferent about hearing from an expert guest lecturer rather than the professor	“*I don't think I would have responded differently.”*	1

*Ten students participated in interviews. Subcodes that were present in a majority (>50%) of interviewee response are highlighted and bolded*.

## Discussion

In January 2020, vaccine hesitancy was widely discussed colloquially and with increasingly robust surveys of various national (41, 46) and international populations (47). These surveys have indicated clear gaps in the general population's understanding of vaccines, virology, and epidemiology (48–50). Moreover, while more research is advancing our understanding of vaccine-hesitancy in some populations (51–53), there remains little understanding of the vaccine plans of non-STEM majors (54) who make up 79% of all awarded bachelor's degrees in the year 2018–2019 (55). The non-majors in this population were demographically similar (see Supplementary Table 1) yet represented two types of students: those going into non-STEM healthcare (e.g., students on pre-nursing track) and those going into neither STEM nor healthcare (e.g., students enrolled in humanities programs). While it is possible students had a range of medical engagement and aspirations, our methods revealed no distinguishable differences between outcomes in the two classes taught in the same modality by the same instructor.

First, we explored the question: How did non-major perceptions of the COVID-19 vaccine change pre to post module?

Our results found that our student population was more likely to perceive the COVID-19 vaccines as both safe and effective following the expert guest lectures intervention (Figure 1). Perceptions of safety and efficacy are linked to overall COVID-19 vaccine acceptance (56, 57). Further, students felt like they could make an informed decision regarding vaccination after the completion of the module (Table 1). Given perception of vaccination (15, 19, 20) and information levels (24, 25) are related to vaccine-acceptance, this would suggest that student's plans to get vaccinated may have shifted due to the module.

Therefore, our second question asked: how did non-majors' plan to get a COVID-19 vaccine change pre to post for our Vaccine Awareness Module? We found that nine surveyed students went from not intending to get vaccinated to intending to get vaccinated (Figure 2). While intentions to take the COVID-19 vaccine may not always manifest follow through (57), declaring intention is often the first step toward vaccination (27). Thus, we established that after the Vaccine Awareness Module, students changed their mind toward vaccination. This led us to the third question: What were non-major students' perceptions of the Vaccine Awareness Module COVID-19 guest lectures? As a field, public health education research seeks to differentiate between effective and ineffective public health educational strategies (58, 59). The strategies employed are often dictated by the population and location of the target audience. Here we evaluated the impact of guest lectures, a common pedagogical tool in higher education (35, 42). While instructors of record are evaluated at the end of the semester at most institutions, evaluation of the impact of guest lectures has been studied only limitedly (35, 39). We had two courses of non-major students at UAB engage with expert guest lectures by a microbiologist, an epidemiologist, and an infectious disease physician. We have previously shown that expert-guest lectures in a non-majors course can impact perceptions of COVID-19 (40). Moreover, it is well-established that the opinions of one's social sphere has the power to supersede other influences (60–62). However, the efficacy of expert guest lectures related to COVID-19 vaccine in a non-major's classroom had not been established. All the 10 interviewees explicitly mentioned they either felt more informed or more assured about getting the COVID-19 vaccines because of the guest lectures (Table 1). Our findings are in line with earlier work that indicates that an audience is receptive to speakers' personal experience (37, 63–65). This implies that even in this period of particularly low scientific trust (33), when given the opportunity to learn from, and pose their questions to scientists, students show trust and acceptance to what scientific experts teach. We note this could have also been influenced by the demographics of the lecturers themselves. The Vaccine Awareness Module was delivered to classes of majority women, many of whom were women of color and included lectures by four women: two white, one south Asian and one Latina woman. While our study did not specifically address the impact of the race or gender of the guest lecturers, previous research suggests that the diverse students are more significantly impacted by diverse guest lecturers (66). As this area needs more research, in future studies, we recommend investigators evaluate the impact of how race, gender, and other identities play a role in student perception of guest lecturers.

**Figure 3:**
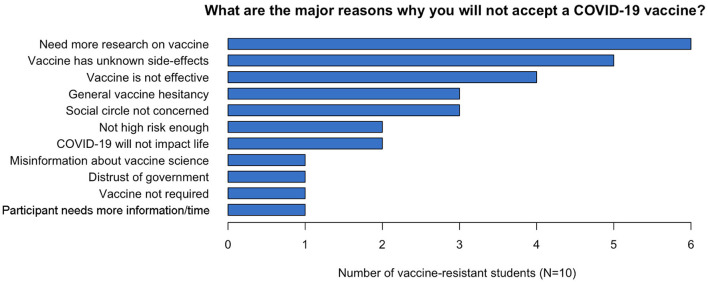
Remaining reasons vaccine-resistant students reject COVID-19 vaccine. Ten post-vaccine awareness respondents who reported not planning to get the vaccine indicated the above reasons for not accepting a COVID-19 vaccine. Ten surveyed respondents answered, “Have you received a COVID-19 vaccine, or do you intend to get a COVID-19 vaccine?” in the post-vaccine awareness questionnaire with “I do not plan to.” The following were the top three remaining reasons why students reported rejecting the vaccines: need for more research (6 of 10), vaccine side effects being unknown (5 of 10), vaccine is ineffective (4 of 10).

Lastly, we wanted to know: what are the remaining reasons vaccine-resistant students reject COVID-19 vaccine? Given the dramatic underutilization of the available healthcare by college-aged individuals (67), we expected that non-major students at the University of Alabama at Birmingham (UAB) would exhibit similar concerns regarding the acceptance of the novel COVID-19 vaccines. Of the 98 respondents to the post Vaccine Awareness Module questionnaire, ten students answered, “Have you received a COVID-19 vaccine, or do you intend to get a COVID-19 vaccine?” with “I do not plan to” (Figure 2). Analysis of the top three reasons why the surveyed students reported rejecting the vaccines included: a perceived need for more research, unknown vaccine side effects, and a belief that the vaccine is ineffective. These responses were reported by six, five and four of the 10 respondents respectively (Figure 3). The persistent concerns expressed by our participants were like those expressed in other studies (41, 56, 57, 68). Further research suggests a link between vaccine reluctance and thoughts about where SARS-COV-2 originated from (69), governmental distrust (70), and belief in non-evidence based theories (26) particularly through social media (28, 29). While not explored here, these beliefs could be underlying the student reasons against vaccination shown in Figure 3 and could thus warrant future research.

The time sensitivity, urgency, and instructor freedom limited the scope of this work in tangible ways. Validated surveys about COVID-19 were not available to us in January 2021 as the vaccines were not made available to the public until Spring 2021. We could not design an ideal experimental design, which would have included cross-comparison design with and without guest lectures, due to course limitations. While our data indicate our intervention directly influenced student perceptions, many other factors may have contributed to students' willingness to get vaccinated during the Spring 2021 semester beyond our intervention. We accounted for these limitations by directly asking students how the instruction impacted them and found that some changes were due to the guest lectures (Table 1).

We suspect that the abrupt transition to online education made students less likely to participate as we previously had much higher participation rates in-person classes at UAB (42). While we cannot say the degree to which these students are representative of the non-majors' populations at large, they mirror demographic composition of the overall UAB student population (71) and nevertheless provide us with insight about non-major's experiences. Much more work is needed to better understand non-major populations, particularly when it comes to COVID-19 vaccine hesitancy. Continued research shows the benefit of connecting everyday issues with non-major students through guest lectures alongside pedagogical interventions like Service-Learning (40, 42, 72). Ongoing work should assess how Service-Learning pedagogies, particularly those using social media (28–30) can impact student perceptions of COVID-19 vaccines. To be able to longitudinally follow the vaccine-resistant students would also uncover meaningful insight that would be useful for better relating and teaching to that population.

Overall, the impact of the expert guest lectures indicates that, as an educational intervention, guest lectures can be impactful in modulating students' vaccination behaviors. Given the increased ubiquity of virtual education, our data suggests that instructors and their students may benefit from expert guest lecturers, especially those pertinent to current affairs and or topics of particular interest to non-major students.

## Data Availability Statement

The raw data supporting the conclusions of this article will be made available by the authors, without undue reservation.

## Ethics Statement

The studies involving human participants were reviewed and approved by University of Alabama Institutional Review Board. The participants provided their written informed consent to participate in this study.

## Author Contributions

CM, SA-J, and SR established the experimental design and research questions. CM coordinated the IRB approval process. CM, SA-J, and SA performed the interviews. OJP validated the interview transcripts. CM, SA-J, and MB contributed to data analysis and figure generation. SA and RF contributed to coding the interview transcripts. CM, SA-J, OJP, BC, DD, MH, and SR contributed to the writing of the first draft of the manuscript and drawing preliminary conclusions. CM, SA-J, MB, and SR edited the manuscript. All authors contributed to the article and approved the submitted version.

## Funding

This material is based upon work supported by the National Science Foundation Research Coordination Networks in Undergraduate Biology Education (Grant No. 1826988 to SR), National Science Foundation Graduate Research Fellowship Program (Grant No. 1450078 to SA-J), and CM is supported by the National Institute of General Medical Sciences training grant (Grant No. K12GM088010 to Lisa Schwiebert).

## Conflict of Interest

The authors declare that the research was conducted in the absence of any commercial or financial relationships that could be construed as a potential conflict of interest.

## Publisher's Note

All claims expressed in this article are solely those of the authors and do not necessarily represent those of their affiliated organizations, or those of the publisher, the editors and the reviewers. Any product that may be evaluated in this article, or claim that may be made by its manufacturer, is not guaranteed or endorsed by the publisher.

## References

[1] Nicola M Alsafi Z Sohrabi C Kerwan A Al-Jabir A Iosifidis C. The socio-economic implications of the coronavirus pandemic (COVID-19): a review. Int J Surg. (2020) 78:185–93. 10.1016/j.ijsu.2020.04.018 32305533 PMC7162753

[2] Walke HT Honein MA Redfield RR. Preventing and responding to COVID-19 on college campuses. JAMA. (2020) 324:1727–8. 10.1001/jama.2020.20027 32991681 PMC9648565

[3] UNESCO. 1.3 Billion Learners Are Still Affected by School or University Closures, as Educational Institutions Start Reopening Around the World, says UNESCO. (2020). Available online at: https://en.unesco.org/news/13-billion-learners-are-still-affected-school-university-closures-educational-institutions (accessed November 16, 2021).

[4] Marinoni G van't Land H. The Impact of COVID-19 on Global Higher Education. International Higher Education, Special Issue 102. Berlin: DUZ Academic Publishers (2020).

[5] Shim TE Lee SY. College students' experience of emergency remote teaching due to COVID-19. Child Youth Serv Rev. (2020) 119:105578. 10.1016/j.childyouth.2020.105578 33071405 PMC7550861

[6] Leidner AJ Barry V Bowen VB Silver R Musial T Kang GJ. Opening of large institutions of higher education and county-level COVID-19 incidence - United States, July 6-September 17, 2020. MMWR Morb Mortal Wkly Rep. (2021) 70:14–9. 10.15585/mmwr.mm7001a4 33411699 PMC7790156

[7] Rennert L Kalbaugh CA Shi L McMahan C. Modelling the impact of presemester testing on COVID-19 outbreaks in university campuses. BMJ Open. (2020) 10:e042578. 10.1136/bmjopen-2020-042578 33323447 PMC7745453

[8] Fadda M Albanese E Suggs LS. When a COVID-19 vaccine is ready, will we all be ready for it? Int J Public Health. (2020) 65:711–2. 10.1007/s00038-020-01404-4 32529534 PMC7288619

[9] CDC. COVID Data Tracker Weekly Review. (2022). Available online at: https://www.cdc.gov/coronavirus/2019-ncov/covid-data/covidview/index.html (accessed November 16, 2021).

[10] BioNTech. BioNTech. Available online at: https://biontech.de/ (accessed November 16, 2021).

[11] Pfizer Inc. Available online at: https://www.pfizer.com/

[12] ModernaTX Inc. Available online at: https://www.modernatx.com/ (accessed November 16, 2021).

[13] Janssen. Janssen: Pharmaceutical Companies of Johnson and Johnson. Available online at: https://www.janssen.com/ (accessed November 16, 2021).

[14] Fontanet A Cauchemez S. COVID-19 herd immunity: where are we? Nat Rev Immunol. (2020) 20:583–4. 10.1038/s41577-020-00451-5 32908300 PMC7480627

[15] Neumann-Bohme S Varghese NE Sabat I Barros PP Brouwer W van Exel J. Once we have it, will we use it? A European survey on willingness to be vaccinated against COVID-19. Eur J Health Econ. (2020) 21:977–82. 10.1007/s10198-020-01208-6 32591957 PMC7317261

[16] El-Mohandes A White TM Wyka K Rauh L Rabin K Kimball SH. COVID-19 vaccine acceptance among adults in four major US metropolitan areas and nationwide. Sci Rep. (2021) 11:21844. 10.1038/s41598-021-00794-6 34737319 PMC8569192

[17] Williams L Gallant AJ Rasmussen S Brown Nicholls LA Cogan N Deakin K. Towards intervention development to increase the uptake of COVID-19 vaccination among those at high risk: outlining evidence-based and theoretically informed future intervention content. Br J Health Psychol. (2020) 25:1039–54. 10.1111/bjhp.12468 32889759

[18] Chemaitelly H Bertollini R Abu-Raddad LJ. Efficacy of natural immunity against SARS-CoV-2 reinfection with the beta variant. N Engl J Med. (2021) 385:2585–6. 10.1056/NEJMc2110300 34910864 PMC8693689

[19] Lin Y Hu Z Zhao Q Alias H Danaee M Wong LP. Understanding COVID-19 vaccine demand and hesitancy: a nationwide online survey in China. PLoS Negl Trop Dis. (2020) 14:e0008961. 10.1371/journal.pntd.0008961 33332359 PMC7775119

[20] Pogue K Jensen JL Stancil CK Ferguson DG Hughes SJ Mello EJ. Influences on attitudes regarding potential COVID-19 vaccination in the United States. Vaccines (Basel). (2020) 8:582. 10.3390/vaccines8040582 33022917 PMC7711655

[21] Abir T Kalimullah NA Osuagwu UL Yazdani DMN Mamun AA Husain T. Factors associated with the perception of risk and knowledge of contracting the SARS-Cov-2 among adults in Bangladesh: analysis of online surveys. Int J Environ Res Public Health. (2020) 17:5252. 10.3390/ijerph17145252 32708161 PMC7400220

[22] Fisher KA Bloomstone SJ Walder J Crawford S Fouayzi H Mazor KM. Attitudes toward a potential SARS-CoV-2 Vaccine: a survey of U.S. adults. Ann Intern Med. (2020) 173:964–73. 10.7326/M20-3569 32886525 PMC7505019

[23] Loomba S de Figueiredo A Piatek SJ de Graaf K Larson HJ. Measuring the impact of COVID-19 vaccine misinformation on vaccination intent in the UK and USA. Nat Hum Behav. (2021) 5:337–48. 10.1038/s41562-021-01056-1 33547453

[24] Motoki K Saito T Takano Y. Scientific literacy linked to attitudes toward COVID-19 vaccinations: a pre-registered study [Brief research report]. Front Commun. (2021) 6:707391. 10.3389/fcomm.2021.707391

[25] Synnott CK. College students' COVID-19 vaccine hesitancy. J Higher Educ Manag. (2021) 36:152–9. 10.2139/ssrn.3770915

[26] Jin Q Raza SH Yousaf M Zaman U Siang JM. Can communication strategies combat COVID-19 vaccine hesitancy with trade-off between public service messages and public skepticism? Experimental evidence from Pakistan. Vaccines. (2021) 9:757. 10.3390/vaccines9070757 34358173 PMC8310328

[27] Pew Research Center. (2019). Trust and Mistrust in Americans' Views of Scientific Experts. Available online at: https://www.newswise.com/pdf_docs/156460235934074_Embargoed%20REPORT%20trust%20scientists%207-30-19.pdf (accessed November 16, 2021).

[28] Puri N Coomes EA Haghbayan H Gunaratne K. Social media and vaccine hesitancy: new updates for the era of COVID-19 and globalized infectious diseases. Hum Vaccines Immunother. (2020) 16:2586–93. 10.1080/21645515.2020.1780846 32693678 PMC7733887

[29] Wilson SL Wiysonge C. Social media and vaccine hesitancy. BMJ Global Health. (2020) 5:e004206. 10.1136/bmjgh-2020-004206 33097547 PMC7590343

[30] Pavić Ž Šuljok A. Vaccination conspiracy beliefs among social science and humanities and STEM educated people—An analysis of the mediation paths. PLoS ONE. (2022) 17:e0264722. 10.1371/journal.pone.0264722 35231050 PMC8887742

[31] Cotner S Thompson S Wright R. Do biology majors really differ from non-STEM majors? CBE Life Sci Educ. (2017) 16:ar48. 10.1187/cbe.16-11-0329 28798210 PMC5589428

[32] Knight JK Smith MK. Different but equal? How nonmajors and majors approach and learn genetics. CBE Life Sci Educ. (2010) 9:34–44. 10.1187/cbe.09-07-0047 20194806 PMC2830160

[33] Kreps S Prasad S Brownstein JS Hswen Y Garibaldi BT Zhang B. Factors associated with US adults' likelihood of accepting COVID-19 vaccination. JAMA Netw Open. (2020) 3:e2025594. 10.1001/jamanetworkopen.2020.25594 33079199 PMC7576409

[34] Paul E Steptoe A Fancourt D. Attitudes towards vaccines and intention to vaccinate against COVID-19: implications for public health communications. Lancet Reg Health Eur. (2021) 1:100012. 10.1016/j.lanepe.2020.100012 33954296 PMC7834475

[35] Merle PF Craig C. Be my guest: a survey of mass communication students' perception of guest speakers. Coll Teach. (2017) 65:41–9. 10.1080/87567555.2016.1232691

[36] Alebaikan RA. Online and face-to-face guest lectures: graduate students' perceptions. Learn Teach Higher Educ. (2016) 13:3–65. 10.18538/lthe.v13.n2.229

[37] Glenwick DS Chabot DR. The undergraduate clinical child psychology course: bringing students to the real world and the real world to students. Teach Psychol. (1991) 18:21–4. 10.1207/s15328023top1801_5

[38] Ricciardelli R Clow KA. The impact of an Exonoree's guest lecture on students' attitudes toward wrongly convicted persons. J Crim Justice Educ. (2012) 23:127–47. 10.1080/10511253.2011.590512

[39] Zheng SL Cheng YS Wang XP Hoffmann C Volkov A. From the source: student-centred guest lecturing in a chemical crystallography class. J Appl Crystallogr. (2018) 51:909–14. 10.1107/S1600576718004120

[40] Adkins-Jablonsky S Fleming R Esteban M Bucio D Morris JJ Raut S. Impacts of a COVID-19 E-service-learning module in a non-major biology course. J Microbiol Biol Educ. (2021) 22:22.1.56. 10.1128/jmbe.v22i1.2489 33884098 PMC8046666

[41] Pew Research Center. (2020). Intent To Get A Covid-19 Vaccine Rises To 60% As Confidence In Research And Development Process Increases. Available online at: https://www.pewresearch.org/science/2020/12/03/covid-19-vaccine-intent-appendix/ (accessed November 16, 2021).

[42] Mendoza DA Adkins-Jablonsky S Bhatt J Morris JJ Raut SA. Service-learning curriculum increases climate change awareness. Sci Educ Civ Engage. (2020) 12:18–20.

[43] Handelsman J Miller S Pfund C. Scientific Teaching. London: Macmillan (2007).

[44] Cho JY Lee E. Reducing confusion about grounded theory and qualitative content analysis: similarities and differences. Qual Rep. (2014) 19:1–20. 10.46743/2160-3715/2014.1028

[45] RStudio Team. (2020). RStudio: Integrated Development for R. Boston, MA: RStudio (2020). Available onine at: http://www.rstudio.com/

[46] Czeisler MÉ Tynan MA Howard ME Honeycutt S Fulmer EB Kidder DP. Public Attitudes, behaviors, and beliefs related to COVID-19, stay-at-home orders, nonessential business closures, and public health guidance — United States, New York City, and Los Angeles, May 5–12, 2020. MMWR Morb Mortal Wkly Rep. (2020) 69:751–8. 10.15585/mmwr.mm6924e1 32555138 PMC7302477

[47] de Figueiredo A Larson HJ. Exploratory study of the global intent to accept COVID-19 vaccinations. Commun Med. (2021) 1:30. 10.1038/s43856-021-00027-x 35602227 PMC9053214

[48] Lewis T. (2021). How the U.S. Pandemic Response Went Wrong—and What Went Right—during a Year of COVID. Available online at: https://www.scientificamerican.com/article/how-the-u-s-pandemic-response-went-wrong-and-what-went-right-during-a-year-of-covid/ (accessed November 16, 2021).

[49] Maxmen A. Why the United States is having a coronavirus data crisis. Nature. (2020) 585:13–4. 10.1038/d41586-020-02478-z 32843755

[50] Weible CM Nohrstedt D Cairney P Carter DP Crow DA Durnova AP. COVID-19 and the policy sciences: initial reactions and perspectives. Policy Sci. (2020) 53:225–41. 10.1007/s11077-020-09381-4 32313308 PMC7165254

[51] Abuown A Ellis T Miller J Davidson R Kachwala Q Medeiros M. COVID-19 vaccination intent among London healthcare workers. Occup Med (Lond). (2021) 71:211–4. 10.1093/occmed/kqab057 34002797 PMC8194640

[52] Townsel C Moniz MH Wagner AL Zikmund-Fisher BJ Hawley S Jiang L. COVID-19 vaccine hesitancy among reproductive-aged female tier 1A healthcare workers in a United States Medical Center. J Perinatol. (2021) 41:2549–51. 10.1038/s41372-021-01173-9 34497336 PMC8424167

[53] Yigit M Ozkaya-Parlakay A Senel E. Evaluation of COVID-19 vaccine acceptance of healthcare providers in a tertiary Pediatric hospital. Hum Vaccin Immunother. (2021) 17:2946–50. 10.1080/21645515.2021.1918523 34018902 PMC8381798

[54] Tam CC Qiao S Li X. Factors associated with decision making on COVID-19 vaccine acceptance among college students in South Carolina. Psychol Health Med. (2021) 27:150–61. 10.1101/2020.12.03.20243543 34555955 PMC8741633

[55] NCES. (2021). Annual Reports: Undergraduate Degree Fields. Available online at: https://nces.ed.gov/programs/coe/indicator/cta (accessed November 16, 2021).

[56] Callaghan TMA Lueck JA Hotez PJ Strych U Dor A Franklin Fowler E. Correlates and disparities of COVID-19 vaccine hesitancy. Soc Sci Med. (2020) 272:113638. 10.1016/j.socscimed.2020.113638 33414032 PMC7834845

[57] Machingaidze S Wiysonge CS. Understanding COVID-19 vaccine hesitancy. Nat Med. (2021) 27:1338–9. 10.1038/s41591-021-01459-7 34272500

[58] Flay BR Burton D. Effective mass communication strategies for health campaigns. In: Atkin C Wallack L editors. Mass Communication and Public Health: Complexities and Conflicts. Newbury Park, CA; London: Sage Publications, Inc. (1990). p. 129–46.

[59] Hou SI. Health education: theoretical concepts, effective strategies and core competencies. Health Promot Pract. (2014) 15:619–21. 10.1177/152483991453804526218487

[60] Castrucci BC Gerlach KK Kaufman NJ Orleans CT. The association among adolescents' tobacco use, their beliefs and attitudes, and friends' and parents' opinions of smoking. Matern Child Health J. (2002) 6:159–67. 10.1023/A:1019774028526 12236663

[61] Costa VB. When science is “another world”: relationships between worlds of family, friends, school, and science. Sci Educ. (1995) 79:313–33. 10.1002/sce.3730790306

[62] Khubchandani J Sharma S Price JH Wiblishauser MJ Webb FJ. COVID-19 Morbidity and mortality in social networks: does it influence vaccine hesitancy? Int J Environ Res Public Health. (2021) 18:9448. 10.3390/ijerph18189448 34574373 PMC8470484

[63] Fulton C. Collaborating in online teaching: inviting e-guests to facilitate learning in the digital environment. Inf Learn Sci. (2020) 121:579–85. 10.1108/ILS-04-2020-0116

[64] Riebe L Sibson R Roepen D Meakins K. Impact of industry guest speakers on business students' perceptions of employability skills development. Industry Higher Educ. (2013) 27:55–66. 10.5367/ihe.2013.0140

[65] Zou P Sun W Hallowell SG Luo Y Lee C Ge L. Use of guest speakers in nursing education: an integrative review of multidisciplinary literature. Adv Med Educ Pract. (2019) 10:175–89. 10.2147/AMEP.S196456 31118860 PMC6497914

[66] Craig CM Bergstrom AM Buschhorn J. All guest speakers are not created equal: diverse students require diverse speakers. J Advertising Educ. (2020) 24:150–67. 10.1177/1098048220956939

[67] Reeves B Rojas-Guyler L Brown J Bennett K Bennett S. Attitudes toward health care needs and utilization of a university health center among LGBT and non-LGBT college students. J Am Coll Health. (2021) 1–9. 10.1080/07448481.2021.1898400 33760704

[68] King WC Rubinstein M Reinhart A Mejia R. Time trends, factors associated with, and reasons for COVID-19 vaccine hesitancy: a massive online survey of US adults from January-May 2021. PLoS ONE. (2021) 16:e0260731. 10.1371/journal.pone.0260731 34932583 PMC8691631

[69] Salali GD Uysal MS. COVID-19 vaccine hesitancy is associated with beliefs on the origin of the novel coronavirus in the UK and Turkey. Psychol Med. (2020) 1–3. 10.1017/S0033291720004067 33070804 PMC7609204

[70] Alcendor DJ. Targeting COVID vaccine hesitancy in rural communities in Tennessee: implications for extending the COVID-19 pandemic in the South. Vaccines. (2021) 9:1279. 10.3390/vaccines9111279 34835210 PMC8621887

[71] UAB. Student Demographics, Full Reports. (2021). Available online at: https://www.uab.edu/institutionaleffectiveness/images/documents/studentdemographics/Undergraduate-Fall-2021.pdf (accessed November 16, 2021).

[72] Strage A. Service-learning as a tool for enhancing student outcomes in a college-level lecture course. Michigan J Commun Serv Learn. (2000) 7:5–13.

