# Supplementary material for: Expert-Led Module Improves Non-STEM Undergraduate Perception of and Willingness to Receive COVID-19 Vaccines
Source: Front Public Health. 2022 May 18;10:816692. doi: 10.3389/fpubh.2022.816692 (PMC9157538; doi:10.3389/fpubh.2022.816692)
Supplement: Supplementary file 2 [file Data_Sheet_1.pdf]

Supplemental Table 2: Demographic description of participants. Comparison of the two courses from which students were recruited for this study during the Spring 2021 semester. (*) Non-white includes Asian, Multiracial, Black, and Hispanic.

Course Title	Course Description	#Participants/ Enrolled	Mean Age (SD)	Female: Male (ratio)	white: Non-white (ratio)*	Underclassmen/ Upperclassmen and other (ratio)
BY101 Topics in Contemporary Biology	Introductory biology for non-majors	43 / 97	20.0 (2.0)	74.4: 5.6	62.8: 37.2	79.1:0.9
BY261 Intro to Microbiology	Introductory microbiology for non-majors	112 / 216	20.6 (3.4)	88.4:11.6	59.8:40.2	62.5:37.5
Combined		155/ 313	20.4 (3.1)	84.5:15.5	94:61	67.1:32.9

Supplemental Box 1. Interview script guide. Based on the questionnaire, the guide provided the structure for the semi-structured interview.

Introduction:

Hi, my name is XXX YYY. I am working with Dr. Raut on her research study on undergraduate perspectives on COVID-19 and its vaccines. At this time, I am going to ask you to rename yourself XXX on Zoom and either turn off or block your webcam. Let me know if you need instructions on how to do that. "You" should now appear as a black box on zoom, with a predetermined "code name". This is to protect your identity. At this time, I am going to hit "record to the cloud"

RECORD.

For the recording, I am XXX YYY and we are here as part of Dr. Raut's research study on the undergraduate perspective on COVID-19 and its vaccines. Today I am interviewing you to hear about your perspective. I expect this interview to take 10-60 minutes. You are free to opt out at any time. Also, as a reminder, your instructor will not know you have agreed to participate, nor will your participation affect any current or future grades. Currently do you consent to participate?

Need an oral yes or no.

We will ask you questions you may have already answered. Please answer as you feel now, even if your responses differ from before. We may also ask you to elaborate on your responses.

1. Have you received a COVID-19 vaccine?

No->Do you intend to get a COVID-19 vaccine?

    Yes -> why haven't you gotten it yet?

        Are you aware that all adults in AL are eligible now and the health department is hosting clinics? You don't need an appointment.

    No -> why?

Yes-> Do you remember which vaccine you got?

    How many doses?

    Can you describe any symptoms you experienced?

    Did those symptoms impact your willingness to get additional doses?

    Would those symptoms affect your willingness to get a booster shot if those become available and necessary?

2. Do/Did you have enough information to decide regarding accepting the COVID-19 vaccine.

Yes -> Can you walk me through the moment when you had enough information?

No ->Since you feel uninformed, what is something that you wish you knew more about?

3. Was it or will it be required by your school or work?

-Would you get a vaccine if school or employer mandated it?

4. Would you say, the coronavirus outbreak is/has been a risk to your personal health?

Yes ->In what ways?

No -> Even though you feel you are not at risk; do you have any responsibility to those that are at risk?

5a. [30 MIN mark]**: Have you or someone you know has been affected by COVID-19?,**

- people in close family or friends?

-first-hand experience with COVID?

5b. How has (knowing/not knowing) someone who has had COVID impacted your willingness to get a COVID-19 vaccine?

6. Do you think the approved COVID-19 vaccines are safe?

-Symptoms

-Where have you learned this info?

7.Do you think the approved COVID-19 vaccines are effective?

-What does effective mean to you?

-What are they effective for?

-Where did you learn this info?

8. Yes vaccine- Why did you accept a COVID-19 vaccine?

No vaccine - Why won't you accept a COVID-19 vaccine?

Name as many reasons as you can think of

9a. BY261 and BY 101: How did the COVID lecture or guest lectures (by Dr. Christina Morra, Dr. Bertha Hidalgo, and Dr. Ellen Eaton) affect your knowledge regarding of COVID-19?

9b. Did the 3 guest lectures affect your knowledge of the approved COVID-19 vaccines?

-Why [did/didn't} the speakers impact your perspective?

-How was hearing from guest lecturers different from hearing from your instructor?

10. What other worries or concerns, if any, do you have regarding COVID-19 vaccination?

11. How did the COVID-19 pandemic (from March 2020 through now), impact your life?

-your schooling (grades, friendships, graduation trajectory)?

-your career plans?

-your social life (religious services, travel plans, friendships, family life)?

12. How will vaccines impact your life?

13. Who do you get information regarding COVID-19 and its vaccines from?

14. Would you like to know or share anything else related to COVID-19 or its vaccines?

At this time, I have no additional questions, so if you have nothing more to ask or share with me, I'd like to thank you for your time in participating in our study. I am now going to stop the recording and end the zoom session.

Complete RStudio Code

```
Book2<-Book2
#confirm variables are numeric
Book2$presafe<-as.numeric(Book2$presafe)
Book2$postsafe<-as.numeric(Book2$postsafe)
Book2$preeffective<-as.numeric(Book2$preeffective)
Book2$posteffective<-as.numeric(Book2$posteffective)
Book2$preinformation<-as.numeric(Book2$preinformation)
Book2$postinformation<-as.numeric(Book2$postinformation)
Book2$premandate<-as.numeric(Book2$premandate)
Book2$postmandate<-as.numeric(Book2$postmandate)
Book2$prepersonalhealth<-as.numeric(Book2$prepersonalhealth)
Book2$postpersonalhealth<-as.numeric(Book2$postpersonalhealth)

#wilcox (Man U) tests (aka nonparametric T-Tests used for likert style data)
wilcox.test(Book2$presafe,Book2$postsafe) #SIGNIFICANT #W = 2768, p-value = 9.56e-06
wilcox.test(Book2$preeffective,Book2$posteffective) #SIGNIFICANT #W = 2688.5, p-value = 3.019e-06
wilcox.test(Book2$preinformation,Book2$postinformation) #SIGNIFICANT #W = 2529.5, p-value = 1.649e-06
wilcox.test(Book2$premandate,Book2$postmandate) #W = 3655, p-value = 0.3388
wilcox.test(Book2$prepersonalhealth,Book2$postpersonalhealth) #W = 4148, p-value = 0.1101

#linear model controlling for class
anova(lm(posteffective~preeffective+class,data=Book2))
#SAFETY SIGNIFICANT p= 0.0001965 ***
#no effect of class p=0.5044434
anova(lm(postsafe~presafe+class,data=Book2))
#EFFECTIVE SIGNIFICANT p = 0.0009106 ***
#no effect of class p = 0.7359697
anova(lm(postinformation~preinformation+class,data=Book2))
#INFORMATION SIGNIFICANT p = 0.02735 *
#no effect of class p = 0.64655
anova(lm(postmandate~premandate+class,data=Book2))
#MANDATE SIGNIFICANT p = 9.786e-05 ***
#no effect of class p = 0.5793
anova(lm(postpersonalhealth~prepersonalhealth+class,data=Book2))
#PERSONAL HEALTH SIGNIFICANT p = 1.293e-07 ***
#no effect of class p = 0.8633

#make variables factors
Book2$presafe<-as.factor(Book2$presafe)
Book2$postsafe<-as.factor(Book2$postsafe)
Book2$preeffective<-as.factor(Book2$preeffective)
Book2$posteffective<-as.factor(Book2$posteffective)
```

```
Book2$preinformation<-as.factor(Book2$preinformation)
Book2$postinformation<-as.factor(Book2$postinformation)
Book2$premandate<-as.factor(Book2$premandate)
Book2$postmandate<-as.factor(Book2$postmandate)
Book2$prepersonalhealth<-as.factor(Book2$prepersonalhealth)
Book2$postpersonalhealth<-as.factor(Book2$postpersonalhealth)

Book2$prestatus<-as.factor(Book2$prestatus)
Book2$poststatus<-as.factor(Book2$poststatus)

#ordinal regression

mod<-polr(postsafe~presafe+class, data=Book2,Hess=T)
summary(mod)
coeffs <- coef(summary(mod))
p <- pnorm(abs(coeffs[, "t value"]), lower.tail = FALSE) * 2
cbind(coeffs, "p value" = round(p,4))
exp(coef(mod))
#no effective of class on post-safe

mod<-polr(posteffective~preeffective+class, data=Book2,Hess=T)
summary(mod)
coeffs <- coef(summary(mod))
p <- pnorm(abs(coeffs[, "t value"]), lower.tail = FALSE) * 2
cbind(coeffs, "p value" = round(p,4))
exp(coef(mod))
#no effect of class on post-effective

mod<-polr(postinformation~preinformation+class, data=Book2,Hess=T)
summary(mod)
coeffs <- coef(summary(mod))
p <- pnorm(abs(coeffs[, "t value"]), lower.tail = FALSE) * 2
cbind(coeffs, "p value" = round(p,4))
exp(coef(mod))
#no effect of class on post-information

mod<-polr(postmandate~premandate+class, data=Book2,Hess=T)
summary(mod)
coeffs <- coef(summary(mod))
p <- pnorm(abs(coeffs[, "t value"]), lower.tail = FALSE) * 2
cbind(coeffs, "p value" = round(p,4))
exp(coef(mod))
#no effect of class on post-mandate

mod<-polr(postpersonalhealth~prepersonalhealth+class, data=Book2,Hess=T)
summary(mod)
coeffs <- coef(summary(mod))
p <- pnorm(abs(coeffs[, "t value"]), lower.tail = FALSE) * 2
cbind(coeffs, "p value" = round(p,4))
exp(coef(mod))
```

```
#no effect of class on post-personalhealth

mod<-polr(poststatus~prestatus+class, data=Book2,Hess=T)
summary(mod)
coeffs <- coef(summary(mod))
p <- pnorm(abs(coeffs[, "t value"]), lower.tail = FALSE) * 2
cbind(coeffs, "p value" = round(p,4))
exp(coef(mod))
#no effect of class on status

#special thanks to https://www.st-
andrews.ac.uk/media/ceed/students/mathssupport/OrdinalexampleR.pdf



------
#making the plot
  library(ggplot2)
library(ggpubr)
theme_set(theme_pubr())

Book2prepost
library(ggplot2)
library(tidyverse)
ggplot(Book2prepost) +
  geom_bar(aes(x = prepost, fill = safe), position = 'fill', alpha = .6,color="black")+
  ylab('Proportion of Students')+
  xlab('')+
  ggtitle('')+
  coord_flip() +
  scale_fill_manual(values =
c("red","brown2","grey","deepskyblue2","deepskyblue4","white")) +
  theme_classic()

Book2prepost
library(ggplot2)
library(tidyverse)
ggplot(Book2prepost) +
  geom_bar(aes(x = prepost, fill = safe), position = 'fill', alpha = .6,color="black")+
  ylab('Proportion of Students')+
  xlab('')+
  ggtitle('')+
  coord_flip() +
  scale_fill_manual(values =
c("red","brown2","grey","deepskyblue2","deepskyblue4","white")) +
  theme_classic()



Book2prepost
library(ggplot2)
```

```
library(tidyverse)
a<-ggplot(Book2prepost) +
  geom_bar(aes(x = prepost, fill = safe), position = 'fill', alpha = .6,color="black")+
  ylab('Proportion of Students')+
  xlab('')+
  ggtitle('The approved COVID-19 vaccines are safe.')+
  theme(plot.title = element_text(hjust = 0.5, size = 20,face =
"bold"),legend.position="none")+
  coord_flip() +
  scale_fill_manual(values =
c("red","brown2","grey","deepskyblue2","deepskyblue4","white"))
a

Book2prepost
library(ggplot2)
library(tidyverse)
b<-ggplot(Book2prepost) +
  geom_bar(aes(x = prepost, fill = effective), position = 'fill', alpha = .6,color="black")+
  ylab('Proportion of Students')+
  xlab('')+
  ggtitle('The approved COVID-19 vaccines are effective.')+
  theme(plot.title = element_text(hjust = 0.5, size = 20,face =
"bold"),legend.position="none")+
  coord_flip() +
  scale_fill_manual(values =
c("red","brown2","grey","deepskyblue2","deepskyblue4","white"))
b

Book2prepost
c<-ggplot(Book2prepost) +
  geom_bar(aes(x = prepost, fill = status), position = 'fill', alpha = .6,color="black")+
  ylab('Proportion of Students')+
  ggtitle('COVID-19 Vaccine Status')+
  theme(plot.title = element_text(hjust = 0.5, size = 20,face =
"bold"),legend.position="none")+
  coord_flip() +
  scale_fill_manual(values = c("snow2","pink","slateblue2","darkslateblue","white"))
c

figure <- ggarrange(a, b,
                    labels = c("A", "B"),
                    ncol = 1, nrow = 2)
figure




#trying to make a different plot
library(ggplot2)
library(reshape2)
```

```
library(RColorBrewer)
library(dplyr)
library(ggthemes)
library(stringr)
tab<-SE
SE$safe<-as.numeric(SE$safe)
SE$effective<-as.numeric(SE$effective)
mytitle<-"The approved COVID-19 vaccines are safe and effective"
#thanks to http://rnotr.com/likert/ggplot/barometer/likert-plots/
mylevels<-c("Strongly disagree", "Disagree", "Neither", "Agree",  "Strongly agree")
numlevels<-length(tab[1,])-1
numcenter<-ceiling(numlevels/2)+1
tab$midvalues<-tab[,numcenter]/2
tab2<-cbind(tab[,1],tab[,2:ceiling(numlevels/2)],
        tab$midvalues,tab$midvalues,tab[,numcenter:numlevels+1])
colnames(tab2)<-c("outcome",mylevels[1:floor(numlevels/2)],"midlow",
            "midhigh",mylevels[numcenter:numlevels])
numlevels<-length(mylevels)+1
point1<-2
point2<-((numlevels)/2)+1
point3<-point2+1
point4<-numlevels+1
mymin<-(ceiling(max(rowSums(tab2[,point1:point2]))*4)/4)*-100
mymax<-(ceiling(max(rowSums(tab2[,point3:point4]))*4)/4)*100
numlevels<-length(tab[1,])-1
temp.rows<-length(tab2[,1])
pal<-brewer.pal((numlevels-1),"RdBu")
pal[ceiling(numlevels/2)]<-"#DFDFDF"
legend.pal<-pal
pal<-c(pal[1:(ceiling(numlevels/2)-1)], pal[ceiling(numlevels/2)],
      pal[ceiling(numlevels/2)], pal[(ceiling(numlevels/2)+1):(numlevels-1)])
tab3<-melt(tab2,id="outcome")
tab3$col<-rep(pal,each=temp.rows)
tab3$value<-tab3$value*100
tab3$outcome<-str_wrap(tab3$outcome, width = 40)
tab3$outcome<-factor(tab3$outcome, levels = tab2$outcome[order(-
(tab2[,5]+tab2[,6]+tab2[,7]))])
highs<-na.omit(tab3[(length(tab3[,1])/2)+1:length(tab3[,1]),])
lows<-na.omit(tab3[1:(length(tab3[,1])/2),])
lows <- lows[rev(rownames(lows)),]

#use likert style
ggplot() + geom_bar(data=highs, aes(x = outcome, y=value, fill=col), position="stack",
stat="identity") +
  geom_bar(data=lows, aes(x = outcome, y=-value, fill=col), position="stack",
stat="identity") +
  geom_hline(yintercept = 2, color =c("white")) +
  scale_fill_identity("Percent", labels = mylevels, breaks=legend.pal, guide="legend") +
  theme_fivethirtyeight() +
  coord_flip() +
```

```
labs(title=mytitle, y="",x="") +
theme(plot.title = element_text(size=14, hjust=0.2)) +
theme(axis.text.y = element_text(hjust=0)) +
theme(legend.position = "bottom")
```
